# Editorial for Special Issue “Bioactive Oxadiazoles”

**DOI:** 10.3390/ijms22083988

**Published:** 2021-04-13

**Authors:** Antonio Palumbo Piccionello

**Affiliations:** Department of Biological, Chemical and Pharmaceutical Sciences and Technologies-STEBICEF, University of Palermo, 90128 Palermo, Italy; antonio.palumbopiccionello@unipa.it

Oxadiazoles are electron-poor, five-membered aromatic heterocycles containing one oxygen and two nitrogen atoms. The oxadiazoles, namely, 1,2,3-, 1,2,4-, 1,2,5-, and 1,3,4-regioisomers, together with N-oxides, benzo-fused, and non-aromatic derivatives, present a wide application range from material science to explosives and bioactive compounds. In the latter field, many possibilities and oxadiazoles are revealed to be active as antitumoral agents, neuroprotective compounds, antimicrobials, antivirals, antidiabetics, etc. This Special Issue on “Bioactive Oxadiazoles” was intended to offer a broad look at the panorama of the potential applications of these compounds toward various diseases. The expectations were met, and many applications of different biologically active compounds were proposed by distinguished researchers. 

The 1,3,4-oxadiazole motif was inserted into two new classes of hybrid compounds, with anti-inflammatory activity, that exert selective COX-2 inhibition [1,2]. This oxadiazole isomer was also included in the structure of indole hybrid able to target Bcl-2, with in vitro pro-apoptotic activity [3]. An interesting bactericidal activity toward Rice disease caused by *Xanthomonas oryzae* is claimed for cinnamic acid derivatives containing the 1,3,4-oxadiazole moiety [4]. Concerning the 1,2,4-oxadiazole isomer, some 2-imidazoline hybrids were reported for their antibacterial and antitumoral activity against pancreas ductal adenocarcinoma [5]. Additionally, benzofurozans were represented in this issue, with the synthesis of novel derivatives as both anticancer and antibacterial compounds [6]. 

The importance of these heterocycles and their applications in many fields was also highlighted by three interesting literature reviews. The antitumoral activity of oxadiazoles was presented with many examples, mainly related to the 1,2,4- and 1,3,4- isomers [7]. Oxadiazoles were recently described as translational readthrough-inducing drugs (TRIDs); this groundbreaking discovery was reviewed in this issue from the employment of *Ataluren* for the treatment of diseases caused by nonsense mutations to recent examples of lead compound’s optimization [8]. Finally, Salassa and Terenzi reviewed the employment of oxadiazoles as a ligand for the construction of metal complexes with interesting applications in the field of material science, antitumoral activity, and fluorescent probes for cell imaging [9].

As Guest Editor of this Special Issue, I would like to acknowledge all the authors for their generous participation and the high scientific value of all the manuscripts, as well as the editorial team at *IJMS* for their support during managing and production of all the submissions.
